# Social and behavior change communication in the fight against malaria in Mozambique

**DOI:** 10.1590/S1518-8787.2017051006360

**Published:** 2017-02-21

**Authors:** Jorge Alexandre Harrison Arroz

**Affiliations:** World Vision Mozambique. Malaria Project Global Funded. Maputo, Moçambique

**Keywords:** Malaria, prevention & control, Social Participation, Health Knowledge, Attitudes, Practice, Evaluation of the Efficacy-Effectiveness of Interventions

## Abstract

Long-lasting insecticide-treated nets and/or indoor residual spraying, associated with case management, are key interventions in the control of malaria in Africa. The objective of this study is to comment on the role of social and behavior change communication as a potential key intervention in the control of malaria in Mozambique.

## RESUMO

As redes mosquiteiras impregnadas com insecticidade de longa duração e/ou pulverização intra-domiciliária, associada ao manejo de casos são intervenções-chave no controlo da malária em África. O objetivo deste estudo foi comentar o papel da comunicação para a mudança social e de comportamento como intervenção potencialmente chave no controlo da malária em Moçambique.

## INTRODUCTION

Malaria is a major public health problem in the world, with approximately 207 million cases and 627,000 deaths per year. Most of the cases (80.0%) and deaths (90.0%) occur in Africa^a^.

Malaria is endemic in Mozambique and it represents 45.0% of all the cases observed in outpatient appointments and 56.0% of the admissions in pediatric wards^b^. The malaria prevalence in children aged six to 59 months is of 35.1%,, and the provinces of Zambezia and Nampula have the highest prevalence (55.2% and 42.2%, respectively), while Maputo City and Maputo Province have the lowest prevalence (2.5% and 4.8%, respectively)^c^.

The use of long-lasting insecticide-treated nets (henceforth referred as “insecticide-treated nets”) and/or indoor residual spraying (henceforth “IRS”) can reduce malaria morbidity and mortality, especially in children and pregnant women^5^ ^,^ ^7^ ^,^ ^a^.

There are three determinants of malaria distribution: i) the epidemiological triad: mosquito, parasite, and human; II) environmental factors; and, III) global factors^11^.

The mosquito, particularly *anopheles gambiae s.s*. and *anopheles funestus*, has a leading role as vector in sub-Saharan Africa and in Mozambique. It is responsible for the sporogonic cycle of the parasite.

The parasite, in particular *plasmodium falciparum*, the most deadly of all the species in sub-Saharan Africa and in Mozambique, and the human being complete the epidemiological triad of the occurrence of the disease^2^ ^,^ ^11^. In the humans, the schizogonic cycle of the parasite is developed. This cycle is subject to the potential of individuals (biologically given or personally acquired), the requirements of life (physiological, psychosocial, and environmental), and the social and environmental determinants.

Among the environmental factors, we highlight: temperature, relative humidity, and rainfall, which promote or inhibit the development of the vector in certain parameters^8^ ^,^ ^11^. In addition to these factors, we mention environmental factors, such as poverty. Malaria is both the cause and effect of poverty^11^. The burden of malaria is high in poor countries, increasingly impoverishing these countries in a vicious circle.

There is a strong negative correlation between the Human Development Index (HDI) and the prevalence of malaria in Mozambique by province, with Pearson coefficient of -0.8 and coefficient of determination of 64.3% (Figure).

**Figure:**
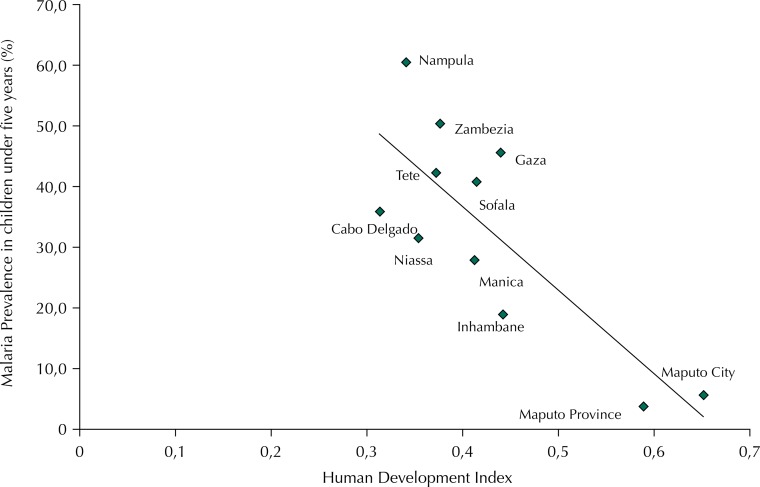
Correlation between malaria prevalence in children under five years and Human Development Index by province. Mozambique, 2011.

Studies and the World Health Organization suggest that insecticide-treated nets and/or IRS associated with case management are key interventions in the fight against malaria^8^ ^,36,^ ^a^ ^,^ ^c^. These studies show that insecticide-treated nets and/or IRS, associated with the appropriate management of malaria cases with appropriate antimalarial drugs, are the best practice and can contribute to the significant reduction in the basic reproductive rate (Ro)^6^ ^,^ ^d^.

The *Ro* of malaria is directly proportional to the vector capacity (C) that is the potential number of secondary cases originating from a primary case, in one day. The term refers to the combination of the components that determine the effectiveness of a local population of mosquitoes in transmitting malaria. Although the human host is the beginning and end of the sequence, the term refers to the stages of the insect, and not the ones that occur in the human host^11^. Therefore, the higher the vector capacity, higher the entomological inoculation rate (EIR) and, therefore, higher *Ro*. The higher *Ro* means high malaria prevalence in a given region.

Vectorial capacity (C) is directly influenced by: i) number of female mosquitoes per person (*m*), II) number of blood intakes per mosquito in one day (*a*), III) efficiency of the malaria transmission system (*b*), IV) possibility of a mosquito becoming infectious (*p* ^n^)^e^.

Taking into account the properties of insecticide-treated nets and/or IRS, we would have the reduction of *m*, *a*, *p*, and *n* and consequently the reduction of C, EIR, Ro, and prevalence of malaria (Table).

**Table:** Key interventions and control of malaria.

Key intervention	Factors affected		
IRS	*m, a, p, n*	C	Ro
Insecticide-treated nets	*m, a, p, n*	C	Ro

a: number of blood intakes per mosquito in one day; m: number of female mosquitoes per person; n: length of sporogony (sexual stage of the life cycle of the *plasmodium* that occurs in the mosquito); p: survival of the mosquito; C: vectorial capacity; Ro: basic reproductive rate

However, such key interventions must consider the social determinants of health (SDOH), the socio-ecological model, and the social and behavior change communication.

According to the Brazilian National Commission on SDOH, SDOH are social, economic, cultural, ethnic/racial, psychological, and behavioral factors that influence the occurrence of health problems and their risk factors in the population. The Commission on Social Determinants of Health of the WHO adopts a shorter definition, according to which SDOH are the social conditions in which persons live and work^4^.

The Dahlgren and Whitehead model about the SDOH establishes four layers of determinants: i) proximal (lifestyles of individuals), ii) social and community networks, iii) living and working conditions, and iv) distal (general socio-economic, cultural, and environmental conditions)^4^. This model establishes that interventions will have greater and better effects when acting on the distal layers.

From the socio-ecological model, the various levels of influence are examined to find the critical point for changes, as well as the individual motivation and knowledge and the social/gender norms, skills, and, favorable environment. This model considers the individual behavior as the product of multiple individual, social, and environmental influences, and it combines the individual change in order to influence the social context in which the individual operates. In this model, the levels of analysis are represented by rings that show the domains of influence and the persons representing them at each level. The first ring, named “individual”, represents the most affected persons. The second and third rings represent the persons who have direct contact with those most affected and who influence their attitudes, beliefs, and actions, shaping the community and gender norms and/or access to, and demand for, community resources and existing services. The fourth ring, the widest, includes those who indirectly influence those most affected by the issue and represents the favorable environment^f^.

Mozambique, as many countries in sub-Saharan Africa, has a high illiteracy rate^g^. Education is found in the layer of “living and working conditions” of the Dahlgren and Whitehead model. An intervention at this level of SDOH can bring greater benefits in the control of malaria.

Social and behavior change communication (SBCC) is an interactive, researched, and planned process that aims to change social conditions and individual behaviors^g^.

The sharing of information about measures of malaria prevention through “social and community networks” (second layer in the Dahlgren and Whitehead model) and rings two and three of the Social-Ecological Model may influence the education of persons and their individual and/or collective lifestyles.

In communities where the engagement in the fight against malaria has been achieved, programs for the control and elimination of malaria can be successful^13^. On the other hand, in communities where the engagement in the fight against malaria has not been achieved, where SBCC has had inappropriate investment, or where economic pressure is high (poverty, famine, food insecurity), vector control interventions for the control and elimination of malaria will not be successful^9^ ^,^ ^10^.

Factors such as exposure to messages, risk perception, self-efficacy, response efficacy, and knowledge on the transmission and symptoms of malaria are associated with preventive behaviors^1^ ^,^ ^3^ ^,^ ^12^.

The use of various approaches can be a synergistic strategy^g^. The only use of health channel (health facilities) does not seem to produce the desired effects, especially in countries where access to health facilities is limited (distance factor) and where they are only used when a woman is pregnant or a child is seriously ill, as in Mozambique^h^. Most persons living in rural communities tend to have a vague involvement with modern medicine and traditional health practitioners and community leaders are often the first line of contact^h^.

Approaches using community radio, pamphlets, lectures, dances, theater, community dialogs, among others, should be considered as synergistic approaches and not as replacements for one or the other. These approaches of SBCC should be a process of mobilization and empowerment of the community, used to provide information, skills, and confidence to communities to gain control over decisions related to their own lives.

The network of community volunteers that make a health committee, in addition to community leaders, volunteers from religious faiths, political parties, traditional health practitioners, all trained in malaria prevention and different approaches of SBCC, can be a precious resource to support health professionals and fortify the key malaria interventions: insecticide-treated nets and/or IRS associated with case management.

Teachers, especially in the primary education, may also be promoters of behavior change when transmitting malaria knowledge to students. This will influence their future behavior toward malaria prevention. On the other hand, students will be able to disseminate information in their communities and households.

Innovative ways to transmit preventive messages on malaria for behavior change should be explored to increase the effectiveness of the synergism of the channels of SBCC.

## FINAL COMMENTS

The provision of key interventions for vector control (insecticide-treated nets and/or IRS) and case management may not be enough to reduce the burden of malaria in Mozambique. Social and behavior change communication can play an important role which might be of similar and/or greater value than vector control. Interventions on social and community networks may be an important point of access for the various methodologies and approaches of social and behavior change communication.

## Footnotes

a World Health Organization. Malaria: Factsheet on the World Malaria Report 2013. Geneva: World Health Organization; 2013 [cited 2015 Apr 27]. Available from: http://www.who.int/malaria/media/world_malaria_report_2013/en/

b Ministério da Saúde (MZ), Direcção Nacional de Saúde Pública. Programa Nacional de Controlo da Malária: Plano Estratégico da Malária 2012-2016. Maputo; 2012. Available from: http://www.rollbackmalaria.org/files/files/countries/mozambique2012-2016.pdf

c Ministério da Saúde (Moçambique), Instituto Nacional de Estatística. Moçambique inquérito demográfico e de saúde 2011. Maputo: Ministério da Saúde; 2013.

d Ro is the potential number of secondary cases that may originate from a primary case, during the entire period of the disease.

e
*p* is the survival of the mosquito, and *n* is the length of sporogony (approximately nine days).

f C-Change. Módulos-C: um pacote de aprendizagem para comunicação para a mudança social e de comportamento. Washington, DC: C-Change; 2011 [cited 2015 Apr 27]. Available from: http://www.thehealthcompass.org/sites/default/files/strengthening_tools/c-moduleportuguese.pdf

g African Library Project. Available from: https://www.africanlibraryproject.org/our-african-libraries/africa-facts

h Malaria Consortium. Diálogos comunitários para crianças saudáveis [Incentivar comunidades a falar]. Outubro, 2012. Available from: http://www.malariaconsortium.org/media-downloads/196
